# Body composition and bone mineral status in patients with Turner syndrome

**DOI:** 10.1038/srep38026

**Published:** 2016-11-30

**Authors:** Kun Shi, Li Liu, Yao-Juan He, Duan Li, Lian-Xiong Yuan, Gendie E. Lash, Li Li

**Affiliations:** 1 Department of Gynecology and Obstetrics, Guangzhou Women and Children’s Medical Center, Guangzhou Medical University, 9 Jingsui Road, Guangzhou, Guangdong, 510160, China; 2 Department of Pediatrics Endocrinology, Guangzhou Women and Children’s Medical Center, Guangzhou Medical University, 9 Jingsui Road, Guangzhou, Guangdong 510160, China; 3 Department of Biostatistics, Sun Yixian University, 74 Zhong Shan Er Road, Guangzhou, Guangdong, 510515, China; 4 Guangzhou Institute of Pediatrics, Guangzhou Women and Children’s Medical Center, Guangzhou Medical University, 9 Jingsui Road, Guangzhou, Guangdong, 510160, China

## Abstract

Turner syndrome (TS) is associated with decreased bone mineral density and increased fracture rate. However, the developmental trajectory of bone density or body composition in patients with TS is still unclear. The present study tested the hypothesis that different karyotypes and/or age contributes to abnormal body composition and decreased bone mineral status parameters in patients with TS. This study included 24 girls with TS, in which 13 girls exhibited X0 karyotype and 11 had mosaicism. Quantitative ultrasound (QUS) assessed the bone mineral status of the calcaneus, including bone mineral density (BMD), amplitude-dependent speed of sound (AD-SOS), broadband ultrasound attenuation (BUA) and InBody 770 assessed body composition. Pearson’s test was performed to correlate measured parameters with patient age. The body composition and bone mineral status parameters were not significantly influenced by patient karyotype. There was a correlation between patient age and AD-SOS (r = −0.61, *P* = 0.002) and BUA (r = 0.50, *P* = 0.013) but not BMD (r = −0.19, *P* = 0.379). In conclusion, there was no effect of karyotype on body composition or body mineral status. Bone mineral status, as evidenced by changes in AD-SOS and BUA, alters with age regardless of karyotype. The developmental trajectory demonstrated in the current study warrants further validation in a longitudinal study.

Turner Syndrome (TS) is the result of the absence or structural abnormality of one copy of the X chromosome and thus is defined as complete or partial absence of one sex chromosome in a phenotypic female, with a birth prevalence of 1/2000 live-born girls^1^. Despite the fact that TS patients attain the same level of education and employment as controls, they report a greater frequency of various medical conditions^2^. Half of the patients with TS have a monosomy X karyotype, whereas mosaicism is identified in the other half^3^. Mosaicism may be associated with a less-severe phenotype, but the degree of mosaicism correlates poorly with phenotype. Girls with TS frequently show features of short stature^4^ and higher fracture susceptibility^5^. The bone fragility observed in patients with TS may be due to X-chromosome abnormalities or estrogen deficiency, and is likely a combination of the two^6^.

Treatment with exogenous recombinant human growth hormone (GH) can effectively increase adult height^7^. Besides GH, hormone replacement treatment (HRT) is also important for girls with TS^1^,^8^,^9^. Timing of introduction of HRT is critical. It is important not to initiate treatment in girls who will have spontaneous puberty. Normally, HRT is initiated at the age of 13 with continuous low-dose conjugated estrogen therapy, and cyclic HRT introduced from the second year^8^. However, both GH and HRT have controversial side effects on bone mineral status. GH was previously found to affect height, body mass percentage and body fat percentage, but did not influence bone mineral density (BMD) or cortical bone thickness^10^. HRT was found to increase spine BMD in a group of 44 TS patients (mean age 43.0 years), while forearm BMD, radius ultra-distal BMD, and hip BMD remained unchanged^11^.

In general GH and HRT are started at different ages and the effect is hard to distinguish with patients of different karyotypes and ages present in the same study. In part this is because the developmental trajectory of bone density or body composition is still unclear. Thus the aim of the current study was to first explore the phenotypic features, body composition and bone mineral status between different karyotypes; monosomy X karyotype or mosaicism. Then the developmental trajectory of bone density or body composition was investigated taking into account karyotype, and GH or HRT treatment. Considering that HRT is normally initiated at the age 13, the patients were again separated into two age groups (below or above 13) to explore the potential age on bone mineral status and body composition.

## Results

### Demographics, anthropometrics, body composition and bone mineral status

In the total group of 24 participants, 13 were diagnosed as karyotype X0 TS and 11 were mosaicism. There was no difference found in terms of age, height, weight, BMI, body water, body protein, body mineral, body lipid, BMD, AD-SOS or BUA between the two karyotype groups (Table 1). The study group was then separated into those <13 years and >13 years of age. Due to the low experimental numbers we were not able to perform the same analysis on the <13 years age group and there was no difference in any of the parameters examined between the two karyotype groups in the >13 years age group (data not shown).

### Comparison of the two age groups (<13 years and >13 years)

In TS patients HRT therapy is started at 13 years of age. To determine whether HRT therapy altered body composition and mineral status the study cohort was separated into two age groups (<13 years and >13 years). AD-SOS increased (P = 0.01) and BUA decreased (P = 0.009) in the >13 years group compared to the <13 years group (Table 2). In addition, body mineral composition (P = 0.05), mineral in bone composition (P = 0.05) and fat mass (P = 0.02) also increased in the >13 years group compared to the <13 years group (Table 3). There was no difference between the two age groups in terms of BMD, body water composition or body protein composition (Tables 2 and 3).

### Influence of age (and HRT) on body composition and bone mineral status

There was no linear correlation between age and levels of body water (r = 0.5, P = 0.06), body protein (r = 0.5, P = 0.06) or body lipid (r = 0.3, P = 0.2). While significant correlations were found between age and body mineral (r = 0.5, P = 0.04) and bone mineral (r = 0.5, P = 0.05) composition the effect of karyotype or HRT therapy on these parameters could not be delineated by regression analysis (data not shown).

**Figure 1:**
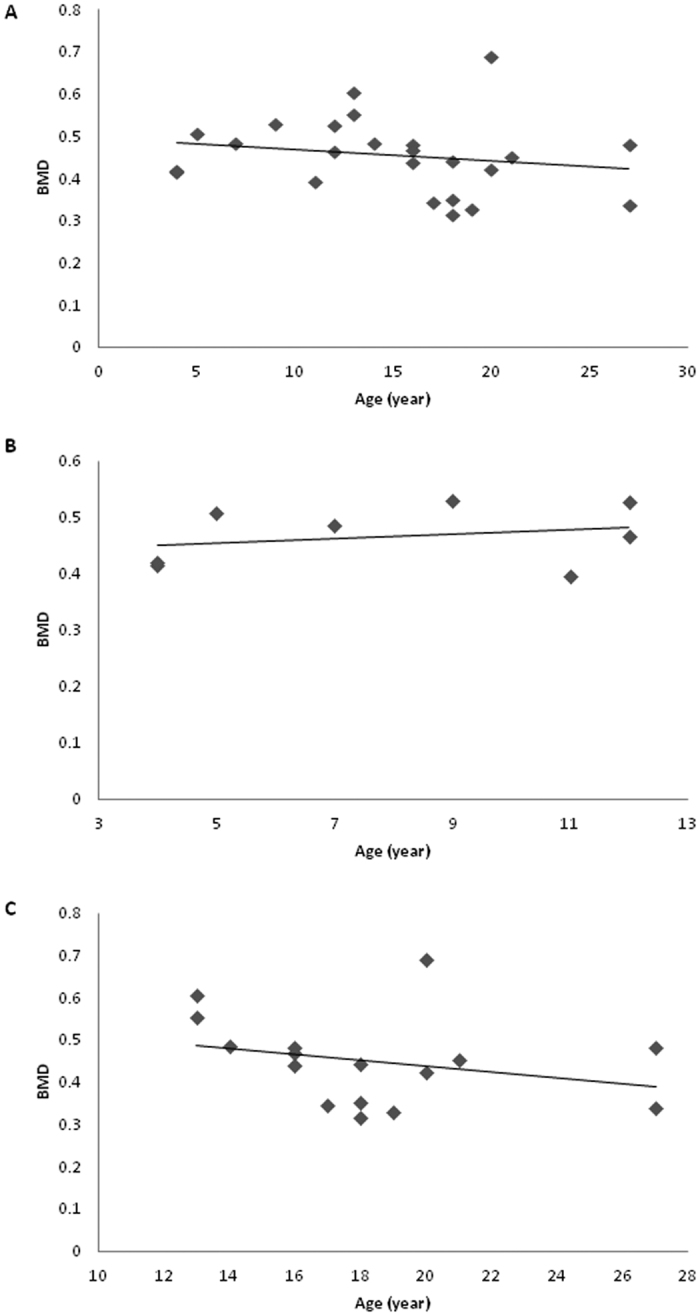
Scatter graphs with lines of best fit showing association between age and BMD (g/cm^2^) measurements in a cohort of TS patients. (**A)** Total cohort (n = 24); (**B)** <13 years of age (n = 8); (**C)** >13 years of age (n = 16).

**Figure 2:**
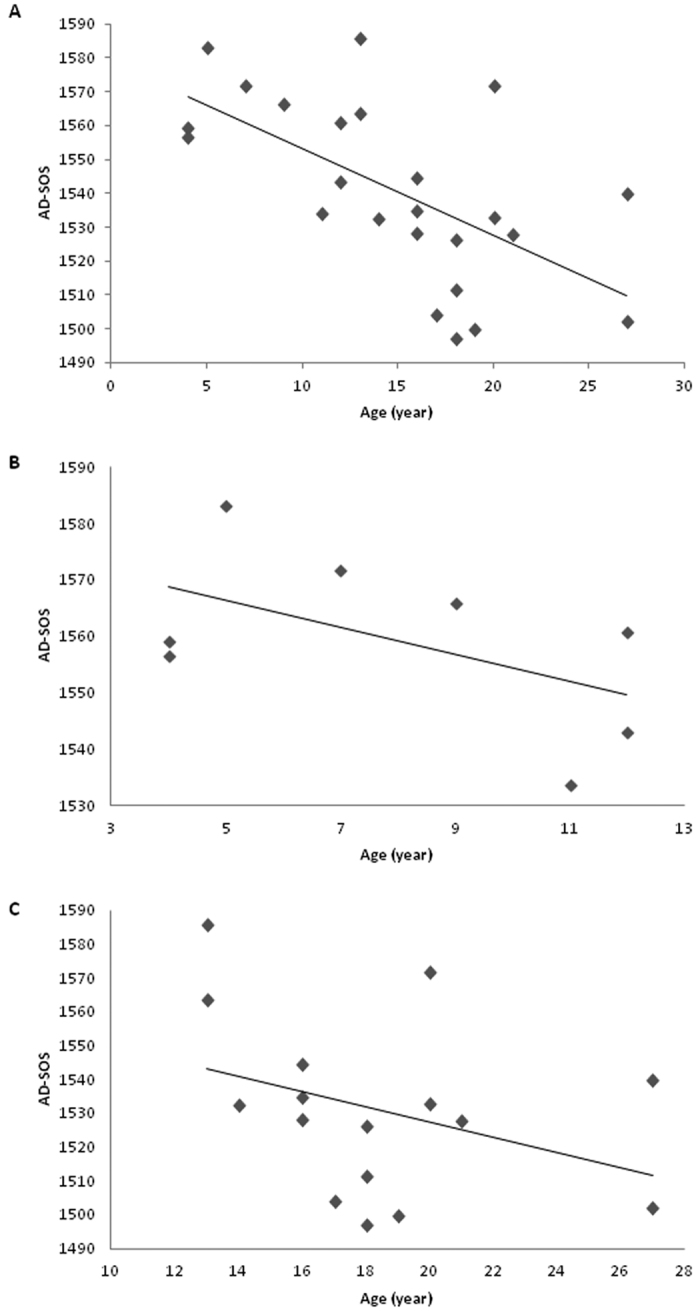
Scatter graphs with lines of best fit showing association between age and AD-SOS (m/s) measurements in a cohort of TS patients. (**A)** Total cohort (n = 24); (**B)** <13 years of age (n = 8); (**C)** >13 years of age (n = 16).

**Figure 3:**
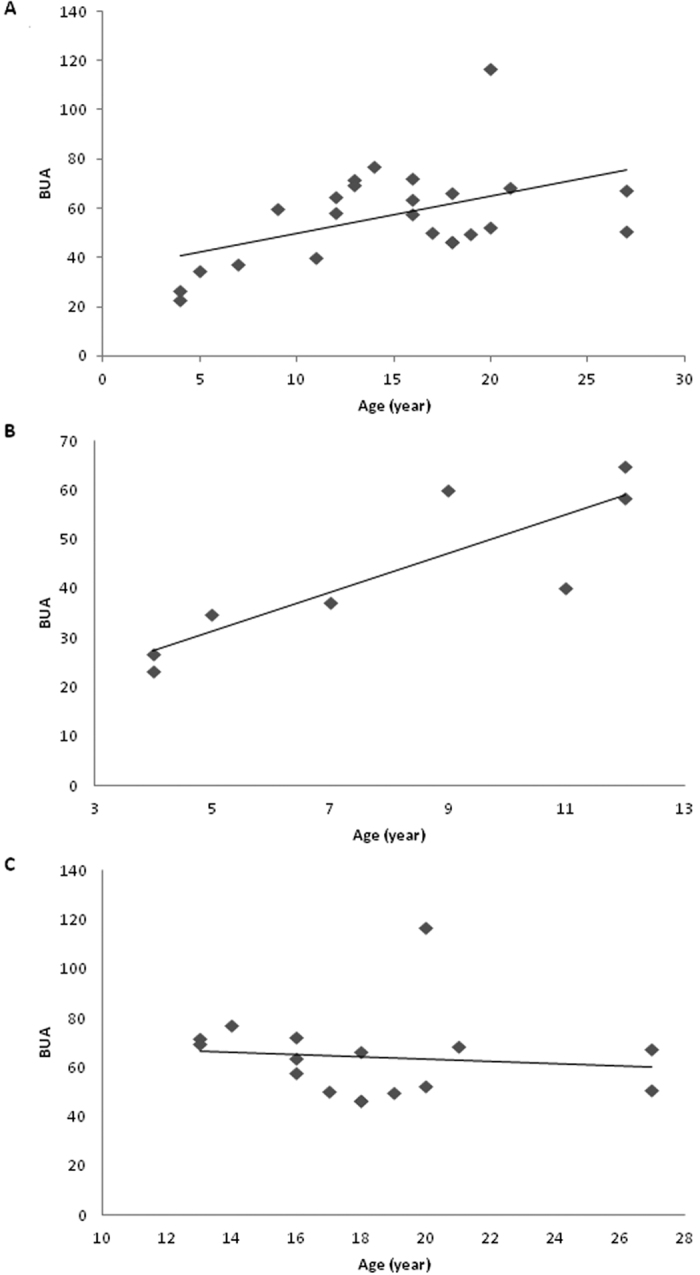
Scatter graphs with lines of best fit showing association between age and BUA (Db/MHZ) measurements in a cohort of TS patients. (**A)** Total cohort (n = 24); (**B)** <13 years of age (n = 8); (**C)** >13 years of age (n = 16).

There was no linear correlation between age and BMD in the total cohort (r = −0.2, P = 0.4; Fig. 1A), or when the patient group was split into those <13 years (Fig. 1B) or >13 years (Fig. 1C), although the slope of these lines differed very slightly (<13 years, 0.004; >13 years −0.007). There was a significant correlation between age and AD-SOS (r = −0.6, P = 0.002, Fig. 2A) and BUA (r = 0.5, P = 0.01, Fig. 3A). For AD-SOS there was no difference in the slope of the line if the analysis was performed for those <13 years (−2.307, Fig. 2B) or those >13 years (−2.360, Fig. 2C). However, the same pattern did not hold true for the BUA measurements (<13 years, 3.898, Fig. 3B; >13 years −0.419, Fig. 3C), suggesting that HRT therapy is unable to overcome the effects of reduced estrogen in TS patients in terms of BUA measurements.

## Discussion

In the current study, we found that there is no difference between different karyotypes for TS (>13years old group) in terms of anthropometric, body composition or body mineral status data. The cross-sectional trajectory showed a linear relationship between age and AD-SOS even taking into account karyotype, or treatment history of GH or HRT. We observed a significant difference in AD-SOS and BUA, but not BMD, in TS patients <13 years old compared to those >13 years old. Interestingly, when studying the slope of the age related curves for AD-SOS and BUA we observed that commencement of HRT therapy did not alter the slope of the curve for AD-SOS but did alter the slope of the curves for BUA which demonstrated an age related increase prior to starting HRT therapy and levels plateauing after HRT therapy.

Although mosaicism may be associated with a less-severe phenotype, the degree of mosaicism correlates poorly with phenotype, likely because of heterogeneity in tissue mosaicism^12^,^13^. In line with previous studies, TS karyotype was found to have no effect on height, weight, BMI, body composition or body mineral status^14^. Recent studies delineated that the short stature in TS is attributed in large part to haplo-insufficiency for the pseudoautosomal gene SHOX, which was found to be responsible for about 2/3 of the height deficit, as well as bone phenotype in TS^15^. However, the heterogeneity in tissue mosaicism or the small sample size may explain the fact that no difference was found between different karyotypes in the current study. Due to the small sample size we were also not able to assess the effect of karyotype on the measured parameters in the group <13 years old, but observed no difference in these parameters in the >13 years age group which was similar to the total cohort. Further investigation into the SHOX gene might help to clarify this relationship.

HRT was found to affect the body composition in healthy elders, but at least part of the known beneficial HRT effects on body composition and bone mass may be regulated by DNA methylation associated alterations in gene expression in circulating white blood cells^16^. The effect of HRT on the body composition in patients with TS remains unclear. We found a significant increase of fat mass, body mineral and body mineral in bone, in patients over 13 years old, after HRT, while the correlation between fat mass and age was absent. In contrast, the fat mass was found unaffected by HRT in a previous study with 45 TS patients^17^. However, there is no difference between the two groups in terms of the other measures of body composition found in the current study, partly because the age range in the current study was restricted to girls below 27 years and the small sample size. Bone fragility in TS patients may also be due to elevated levels of FSH in adolescence prior to HRT administration. Increased FSH levels have been associated with enhanced osteoclastogenesis, although this was also observed post HRT administration in a mechanism associated with increased RANKL expression^18^. These data may suggest why variable success on bone fragility and different bone density measurements are observed even after HRT administration^18^.

In healthy adolescents, cortical bone BMD was found to increase with age, with higher annual accrual rates in girls compared to boys^19^. The current study demonstrated a different pattern in girls with TS, with no significant increase in BMD with increasing age and HRT did not appear to alter the rate of change in BMD with age. In a case-control study of bone density and size, a positive correlation between age and vertebral bone density was present in the control group while there was a negative correlation in prepubertal patients with TS^20^. However, the current study was performed with QUS of the calcaneal bone and a recent study demonstrated that it is difficult to compare data between studies using different measurement methods or bone locations^21^, which may account for variations in the current study compared to others.

AD-SOS and BUA are thought to be more sensitive in detecting the bone mineral status and fracture risk in girls with TS than BMD alone^22^. AD-SOS is a measure of the speed at which the ultrasound of a particular amplitude passes through the bone and soft tissue and reflects the bone architecture and elasticity^23^. While BUA is a measure of how the ultrasound signal is absorbed or scattered by the bone and therefore reflects its porosity (density) without taking into account the surrounding soft tissue^23^. In healthy girls, BUA has been shown to increase with age^23^,^24^ while data for AD-SOS is less consistent with one study showing an increase^24^, and another no change^23^. In our cohort of TS patients BMD did not alter with age, AD-SOS decreased and BUA increased. These data suggest that bone characteristics were altered in TS patients in a manner differing from healthy controls (although this group was not measured in our study and we are basing this conclusion from data in the literature). The reason for this distribution might be that BUA was found to be more sensitive to weight and vitamin D status^25^, while AD-SOS in elder women showed stronger negative correlation with age, who present lower estrogen levels, like TS patients. Furthermore, alterations in both BUA and AD-SOS measurements have different genetic and environmental risk factors^6^,^26^,^27^,^28^.

HRT is frequently used in treating TS, although its effect on bone mineral status remains controversial. According to a questionnaires’ survey, HRT is used in 83% of TS patients, while GH is used in only 37% of patients^29^. Depending on timing of diagnosis and economic status some patients might receive GH treatment prior to onset of HRT, while others may receive no treatment at all due to controversy surrounding the side effects of HRT. Hence, studies investigating the potential effectiveness of HRT in treatment of TS patients are still required. Timing of introduction of HRT is critical and is normally initiated at the age of 13 after spontaneous puberty. Thus the age 13 may mark the turning point of bone or body development. In a study of 32 girls with TS, no significant association was found between apparent bone mineral density and karyotype, growth hormone or timing of oestrogen therapy^30^. While in another study which compared patients on HRT with different on-set ages demonstrated that HRT increased BMD especially in the early onset age group (onset <18 years)^9^. In our cohort of TS patients we demonstrated that BUA also increased with age, although there was only a linear relationship in those girls <13 years old. In contrast, AD-SOS values decreased with age at a similar rate in those <13 years old and those >13 years of age. Therefore, it appears that HRT therapy has no influence on bone architecture and elasticity but is unable to overcome the effects of diminished estrogen levels on bone porosity. A larger study is required to fully delineate the effects of age and HRT therapy on these bone parameters and their usefulness within the clinical setting to assess those TS patients at higher risk of fractures.

## Conclusion

We found no effect of karyotype on body anthropometric, body composition or body mineral status data. AD-SOS changes along with age in regardless of karyotype, GH or HR treatments, while BUA measurements appear to be influenced by HRT. Future study in a larger sample size or longitudinal design would help to further demonstrate the relationship. AD-SOS might be more sensitive and promising in serving as a marker in monitoring the bone health in patients with TS in follow-up visits. By paying early attention to bone mineral status preventative action can be taken, for example nutrient supplements (e.g. calcium, vitamin D) can be given, as well as advice on lifestyle and exercise choices. In addition, in depth investigation of the SHOX gene would also add to the current conclusion.

## Methods

### Participants

A total of 24 Chinese girls with TS (mean age 15.0 ± 6.28 years), were recruited from the outpatient clinic in the Divisions of Pediatric Endocrinology and Gynecology Endocrinology of the Guangzhou Medical University, Guangzhou Women and Children’s Medical Center, China. The diagnosis of TS was made by karyotyping, in which 13 girls exhibited X0 karyotype and 11 had a mosaicism (45X/46XX (n = 9), 46XX with major deletion or isochromosome (n = 2)). None of the participants was affected by any other disease known to be associated with abnormal bone mineral status. Nine girls had been treated with GH, with a dose of 0.330–0.375 mg/kg/week, with 5 of them ceasing treatment as they reached their final height. None of the girls had spontaneous puberty. All girls (aged >13 years) with primary amenorrhea had HRT. HRT was started with continuous low-dose conjugated estrogen therapy (0.5 mg daily for first 6 months, continued with 1 mg daily for another 6 months), and cyclic HRT was initiated from the second year (conjugated estrogens 1 mg/d for 21 days, adding progesterone 10 mg/day for 10 days). The study was approved by the ethics committee for human investigation at Guangzhou Women and Children’s Medical Center. The study was performed in accordance with the approved guidelines and written informed consent was obtained from the participants enrolled or the parents of those with a chronologic age below 18.

### Anthropometry

In all subjects standing height and weight were measured with a wall-mounted stadiometer and a mechanical balance. In each patient, the measurements of both height and weight were repeated three times to arrive at a mean result. Body mass index (BMI) was calculated as weight (kg) divided by height (m) squared.

### Bone Mineral Status

Bone mineral status was assessed with measurements at the calcaneus, including bone mineral density (BMD, g/cm^2^), amplitude-dependent speed of sound (AD-SOS, m/s), broadband ultrasound attenuation (BUA, Db/MHZ) by quantitative ultrasound (QUS, Hologic Inc., Sahara). This device consists of two unfocused transducers mounted coaxially on a monitor caliper. One transducer acts as the transmitter, the other as a receiver. The transducers are acoustically coupled to the heel using soft rubber pads and an oil-based coupling gel. The device calculates the speed of sound (m/s) through the phalanx by dividing the width of the calcaneus (including soft tissues) by the time of flight, which was defined as the time from emitting pulse to receiving signal, taking into account only the signal which reaches a predetermined minimum amplitude value (2 mV) for the first time. Thus, the assessed ultrasound velocity is amplitude-dependent (AD-SOS). Bone and soft tissue alter the acoustic absorption and scattering of the ultrasonic energy signal leading to its attenuation (BUA). BUA is calculated by averaging the pulses sent and received and feeding them in to a least-squares linear model. QUS can precisely reflect the bone structure and bone mechanical properties (strength, stiffness of bone, elastic, porosity, etc.) which can’t be measured by other conventional skeletal radiography, radiographic photo densitometry, dual-energy X-ray absorption metry (DXA), or quantitative computed tomography (QCT). In addition to QUS is known to be safe, radiation free and relatively inexpensive compared with DXA and QCT which are not used in clinical studies.

Body composition such as total body water, protein, minerals, lipid were acquired using an multi-frequency bioelectrical impedance analyzer InBody 770 scanner (In-body Bldg, Seoul, Korea), with high resolution touch screen, frequency 1,5, 50, 260, 500, 1000 kHz and measurement time 60 seconds, with the subjects in a standing position according to the manufacturer’s instructions after shoes, coats and sweaters had been removed.

### Statistical analysis

The data are reported as mean ± SD. Scatter plot and correlation analysis (Pearson’s correlation coefficients) were performed to reveal the possible correlation between bone mineral status, body composition and age of patients. Differences of bone mineral status, body composition between different age groups or karyotypes were tested by unpaired *t*-test. All statistical analyses were carried out using the SPSS (Statistical Package of Social Sciences, Chicago, IL, USA) for Windows software program version 19.0. A *P* value <0.05 was considered statistically significant.

## Additional Information

**How to cite this article**: Shi, K. *et al*. Body composition and bone mineral status in patients with Turner syndrome. *Sci. Rep.* **6**, 38026; doi: 10.1038/srep38026 (2016).

**Publisher's note:** Springer Nature remains neutral with regard to jurisdictional claims in published maps and institutional affiliations.

## Figures and Tables

**Table 1:** Demographic, anthropometric, body composition and bone mineral status (mean ± SD) in participants with TS (n = 24) with different karyotype status.

	Total (n = 24)	Karyotype (45 × 0) (n = 13)	Mosaicism (n = 11)	*P* value
Age (year)	15.0 ± 6.28	13.8 ± 6.7	16.3 ± 5.75	0.3
Height (cm)	135.3 ± 17.19	131.8 ± 19.64	139.2 ± 15.53	0.3
Weight (kg)	35.0 ± 13.19	32.8 ± 15.58	37.5 ± 9.79	0.4
BMI (kg/m^2^)	20.0 ± 4.33	21.6 ± 6.00	18.8 ± 2.30	0.3
Body Water*	19.9 ± 4.11	19.1 ± 5.53	20.7 ± 2.39	0.5
Body Protein*	5.3 ± 1.10	5.1 ± 1.48	5.5 ± 0.62	0.5
Body Mineral*	1.9 ± 0.45	1.84 ± 0.61	1.98 ± 0.26	0.6
Mineral in Bone*	1.6 ± 0.37	1.53 ± 0.49	1.68 ± 0.23	0.4
Fat mass	12.0 ± 6.64	13.3 ± 8.24	10.9 ± 5.08	0.5
BMD	0.46 ± 0.09	0.46 ± 0.08	0.45 ± 0.11	0.8
AD-SOS	1540 ± 26.38	1534.7 ± 27.91	1545.5 ± 24.4	0.3
BUA	57.2 ± 19.37	60.7 ± 16.05	54.2 ± 22.98	0.4

^*^Body composition data was only available on 17 out of the 24 participants, including 8 45 × 0 and 9 mosaicism.

**Table 2:** Bone mineral status in TS patients before and after commencement of HRT therapy (mean ± SD).

	<13 years old (n = 8)	≥13 years old (n = 16)	*P* value
BMD	0.47 ± 0.05	0.45 ± 0.10	0.7
AD-SOS	1559.3 ± 15.56	1531.2 ± 25.97	0.01
BUA	43.2 ± 15.85	64.2 ± 17.33	0.009

**Table 3:** Body composition in TS patients before and after commencement of HRT therapy (mean ± SD).

	<13 years old (n = 3)	≥13 years old (n = 14)	*P* value
Body Water	16.2 ± 4.46	20.7 ± 3.73	0.08
Body Protein	4.3 ± 1.19	5.6 ± 0.99	0.08
Body Mineral	1.5 ± 0.41	2.0 ± 0.40	0.05
Mineral in Bone	1.2 ± 0.37	1.7 ± 0.33	0.05
Fat mass	4.0 ± 0.92	13.7 ± 6.01	0.02

## References

[b1] Ari M., Bakalov V. K., Hill S. & Bondy C. A. The effects of growth hormone treatment on bone mineral density and body composition in girls with turner syndrome. J Clin Endocrinol Metab. 91, 4302–5 (2006). 16940444 10.1210/jc.2006-1351

[b2] Naess E. E., Bahr D. & Gravholt C. H. Health status in women with turner syndrome: a questionnaire study on health status, education, work participation and aspects of sexual functioning. Clin Endocrinol (Oxf). 72, 678–84 (2010). 19769615 10.1111/j.1365-2265.2009.03715.x

[b3] Levitskya L. L. . Turner syndrome: update on biology and management across the life span. Wolters Kluwer Health 22, 65–72 (2015). 10.1097/MED.0000000000000128 25517026

[b4] Gravholt C. H. . Increased fracture rates in turner’s syndrome: a nationwide questionnaire survey. Clin Endocrinol (Oxf). 59, 89–96 (2003). 12807509 10.1046/j.1365-2265.2003.01807.x

[b5] Soucek O. . Bone geometry and volumetric bone mineral density in girls with Turner syndrome of different pubertal stages. Clin Endocrinol. (Oxf). 74, 445–52 (2011). 21138463 10.1111/j.1365-2265.2010.03955.x

[b6] Faienza M. F. . Bone Fragility in Turner Syndrome: Mechanisms and Prevention Strategies. Front Endocrinol (Lausanne) 7, 34 (2016). 27199891 10.3389/fendo.2016.00034 PMC4844601

[b7] Menke L. A. . Efficacy and safety of oxandrolone in growth hormone-treated girls with turner syndrome. J Clin Endocrinol Metab. 95, 1151–60 (2010). 20061421 10.1210/jc.2009-1821

[b8] Trolle C., Hjerrild B., Cleemann L., Mortensen K. H. & Gravholt C. H. Sex hormone replacement in turner syndrome. Endocrine. 41, 2200–9 (2012). 10.1007/s12020-011-9569-8 22147393

[b9] Nakamura T. . Efficacy of estrogen replacement therapy (ERT) on uterine growth and acquisition of bone mass in patients with Turner syndrome. Endoc J. 62, 965–70 (2015). 10.1507/endocrj.EJ15-0172 26289838

[b10] Lo J. Does growth hormone therapy benefit body composition and glucose homeostasis in girls with Turner syndrome? Nat Clin Pract Endocrinol Metab. 4, 596–7 (2008). 18725904 10.1038/ncpendmet0954

[b11] Cleemann L. . Long-term hormone replacement therapy preserves bone mineral density in turner syndrome. Eur J Endocrinol. 161, 251–7 (2009). 19447901 10.1530/EJE-09-0020

[b12] Radtke A. C., Sauder C., Rehm J. L. & McKenna P. H. Complexity in the diagnosis and management of 45, X Turner Syndrome mosaicism. Urology. 84, 919–21 (2014). 25260452 10.1016/j.urology.2014.06.030

[b13] Zhong Q. & Layman L. C. Genetic considerations in the patient with Turner syndrome–45, X with or without mosaicism. Fertil Steril. 98, 775–9 (2012). 23020909 10.1016/j.fertnstert.2012.08.021 PMC3573687

[b14] El-Mansoury M. . Chromosomal mosaicism mitigates stigmata and cardiovascular risk factors in Turner syndrome. Clin Endocrinol (Oxf). 66, 744–51 (2007). 17381484 10.1111/j.1365-2265.2007.02807.x

[b15] Soucek O. . Prepubertal girls with Turner syndrome and children with isolated SHOX deficiency have similar bone geometry at the radius. J Clin Endocrinol Metab. 98, 1241–7 (2013). 10.1210/jc.2013-1113 23666967

[b16] Bahl A. . Hormone Replacement Therapy Associated White Blood Cell DNA Methylation and Gene Expression are Associated With Within-Pair Differences of Body Adiposity and Bone Mass. Twin Res Hum Genet. 18, 647–61 (2015). 26678050 10.1017/thg.2015.82

[b17] Gabel L. . Reexamining the Surfaces of Bone in Boys and Girls During Adolescent Growth: A 12-Year Mixed Longitudinal pQCT Study. J Bone Miner Res. 30, 2158–67 (2015). 26058373 10.1002/jbmr.2570 PMC5059154

[b18] Faienza M. F. . Mechanisms of enhanced osteoclastogenesis in girls and young women with Turner’s Syndrome. Bone. 81, 228–36 (2015). 26208797 10.1016/j.bone.2015.07.021

[b19] Qi W., Li S., Shen Q., Guo X. & Rong H. Effects of recombinant human growth hormone therapy on carbohydrate, lipid and protein metabolisms of children with Turner syndrome. Pak J Med Sci. 30, 731–34 (2014). 25097506 10.12669/pjms.304.4546 PMC4121687

[b20] Pitukcheewanont P. . Bone size and density measurements in prepubertal children with turner syndrome prior to growth hormone therapy. Osteoporos lnt. 22, 1709–15 (2011). 10.1007/s00198-010-1375-2 20827549

[b21] Nadeem M. & Roche E. F. Bone health in children and adolescent with Turner syndromeJ Pediatr Endocr Met. 25, 823–33 (2012). 10.1515/jpem-2012-0088 23426807

[b22] Vierucci F. . Usefulness of phalangeal quantitative ultrasound in identifying reduced bone mineral status and increased fracture risk in adolescents with Turner syndrome. Hormones (Athens). 13, 353–60 (2014). 25079459 10.14310/horm.2002.1485

[b23] Baroncelli G. I. Quantitative ultrasound methods to assess bone mineral status in children: technical characteristics, performance, and clinical application. Pediatr Res. 63, 220–28 (2008). 18287958 10.1203/PDR.0b013e318163a286

[b24] Lee M. . Longitudinal changes in calcaneal quantitative ultrasound measures during childhood. Osteoporos lnt. 22, 2295–305 (2011). 10.1007/s00198-010-1458-0 PMC3988661 20976593

[b25] Maggi S. . Quantitative ultrasound calcaneous measurements: normative data for the Italian population. the ESOPO study. J Clin Densitom. 10, 340–6 (2007). 17470406 10.1016/j.jocd.2007.03.099

[b26] Lee M. . Genome-wide linkage scan for quantitative trait loci underlying normal variation in heel bone ultrasound measures. J Nutr Health Aging. 16, 8–3 (2012). 22237995 10.1007/s12603-011-0080-y PMC3928037

[b27] Williams L. J. . Quantitative Heel Ultrasound (QUS) measures of bone quality in association with mood and anxiety disorders. J Affect Disord. 25, 146, 395–400 (2013). 10.1016/j.jad.2012.09.025 23122528

[b28] Sohl E., de Jongh R. T., Swart K. M., Enneman A. W. & van Wijngaarden J. P. The association between vitamin D status and parameters for bone density and quality is modified by body mass index. Calcif Tissue Int. 96, 113–22 (2015). 25539856 10.1007/s00223-014-9943-7

[b29] Gravholt C. H. . Increased fracture rates in turner’s syndrome: a nationwide questionnaire survey. Clin Endocrinol (Oxf). 59, 89–96 (2003). 12807509 10.1046/j.1365-2265.2003.01807.x

[b30] Przepiera E. S. . Association between ER-a polymorphisms and bone mineral density in patients with Turner syndrome subjected to estroprogestagen treatment—a pilot study. J Bone Miner Metab. 29, 484–492 (2011). 21271267 10.1007/s00774-010-0247-3

